# Rumen fermentation and performance of Hanwoo steers fed total mixed ration with Korean rice wine residue

**DOI:** 10.1186/s40781-016-0084-6

**Published:** 2016-01-11

**Authors:** Chang-Dae Jeong, Lovelia L. Mamuad, Jong Youl Ko, Ha Guyn Sung, Keun Kyu Park, Yoo Kyung Lee, Sang-Suk Lee

**Affiliations:** Ruminant Nutrition and Anaerobe Laboratory, Department of Animal Science and Technology, College of Bio-industry Science, Sunchon National University, 413 Jungang-ro, Suncheon, Jeonnam 57922 Republic of Korea; National Agricultural Cooperative Federation, Anseong-si, Gyeonggi-do, 456-824 South Korea; Adbiotech Co. Ltd., Chun-Cheon City, 200-880 Republic of Korea; Animal Resources Research Center, School of Animal Life and Science, Konkuk University, 120 Neungdong-ro, Gwangjin-gu, Seoul, 05029 South Korea; Rural Development Administration, National Institute of Animal Science, Wanjugun, Jeollabukdo, South Korea

**Keywords:** By-products, Hanwoo steers, In vitro, Korean rice wine residue

## Abstract

**Background:**

This study was conducted to evaluate the effects of adding Korean rice wine residue (RWR) in total mixed ration (TMR) on in vitro ruminal fermentation and growth performance of growing Hanwoo steers.

**Methods:**

For in vitro fermentation, the experimental treatments were Control (Con: 0 % RWR + TMR), Treatment 1 (T1: 10 % RWR + TMR), and Treatment 2 (T2: 15 % RWR + TMR). The rumen fluid was collected from three Hanwoo steers and mixed with buffer solution, after which buffered rumen fluid was transferred into serum bottles containing 2 g dry matter (DM) of TMR added with or without RWR. The samples were then incubated for 0 h, 12 h, 24 h, or 48 h at 39 °C and 100 rpm. For the in vivo experiment, 27 Hanwoo steers (6 months old) with an average weight of 196 ± 8.66 kg were subjected to a 24-week feeding trial. The animals were randomly selected and equally distributed into three groups. After which the body weight, feed intake and blood characteristics of each group were investigated.

**Results:**

The pH of the treatments decreased significantly relative to the control during the 12 h of incubation. Total gas production and ammonia nitrogen (NH_3_-N) was not affected by RWR addition. The total volatile fatty acid (VFA) was lower after 24 h of incubation but at other incubation times, the concentration was not affected by treatments. Feed cost was 8 % and 15 % lower in T1 and T2 compared to control. Blood alcohol was not detected and a significant increase in total weight gain and average daily gain were observed in Hanwoo steers fed with RWR.

**Conclusion:**

Overall, the results of this study suggest that TMR amended with 15 % RWR can be used as an alternative feed resource for ruminants to reduce feed cost.

## Background

Over the last decade, cattle feed costs have increased by up to 70–80 %. This has caused a burden to Korean farmers (Korea Feed Association, 2014). Accordingly, there is now a demand for low cost alternative feedstuffs. One possible alternative is the by-products. By products are derived from food processing and manufacturing that cannot be used as food for humans, but often contains high nutrients and are fairly inexpensive. Food manufacturing by-products are distiller’s dried grains with solubles, tofu cake, rice bran, and wet green tea waste. They are high in crude protein (CP), fatty acids, tannins, and vitamins [1] and therefore have been great potential for utilization as animal feed. For example, corn distillers dried grain with soluble diets have replaced up to 40 % of total feed as a protein source in the United States [2] and the use of wet distillers grain (WDG) up to 10 % in total mixed ration (TMR) did not show any negative effect on the performance of Hanwoo steers [3].

In search for alternative feed, we found out that there are 681 Korean traditional rice wine (Makgeolli) processing companies in South Korea. These companies annually produce about 83,808 tons of Korean rice wine residues (RWR). Korean rice wine residues contain high protein and energy source [4]. However, RWR has high moisture content of about 60–70 %. Also, it is currently not used by agricultural sector due to insufficient data regarding its characteristics and effects. And as a result, rice wine manufacturing companies usually dispose RWR by burning or landfill, which causes environmental problems. In view of these economic and environmental concerns, strategies to efficiently utilize industrial by-products are necessary. Thus, the objective of this study was to evaluate the effects of wet TMR including rice wine residue as replacement for concentrate on in vitro ruminal fermentation and growth performance of growing Hanwoo steers.

## Methods

### Experimental feed and treatments

Korean rice wine residue has an average DM, CP, EE, CF, ash, and NFE contents of 49 %, 9.18 %, 0.66 %, 0.88 %, 0.35 %, and 37.07 %, respectively. Korean rice wine residue was drained and stabilized for 48 h prior to incorporation into the animal feeds. Total mixed ration feed was formulated based on the standard tables of feed composition provided by the Korean National Institute of Animal Science (2007). The composition, nutrient content and feed cost of the feed used in each treatment in the present study are provided in Table 1. The chemical compositions were analyzed according to the guidelines of the Association of Official Analytical Chemists [5], while neutral detergent fiber (NDF) and acid detergent fiber (ADF) were measured using an Ankom fiber analyzer (Ankom Tech. Corp, Fairport, NY, USA) based on the method described by Van Soest et al. (1991). The experimental feeds consisted of a control (non-addition of RWR), Treatment 1 (feed containing RWR at 10 %), or Treatment 2 (feed containing RWR at 15 %) on a percent DM basis.

**Table 1:** Composition, nutrient content and feed cost of total mixed ration containing Korean rice wine residue (RWR) at different inclusion rates for Hanwoo steers at growing stage

Parameters	Treatments		
	Con	T1	T2
Feed composition(% of DM)			
Oat hay	41.32	38.17	36.26
Timothy hay	14.75	13.63	13.79
Alfalfa hay	4.77	4.41	4.46
Wheat grain	4.78	3.19	3.23
Corn	18.34	15.72	15.91
Corn gluten feed	3.34	3.12	3.16
Lupine	6.63	6.13	6.20
Brewers grain	4.13	3.82	0.00
Salt	0.28	0.26	0.26
Limestone	0.58	0.53	0.54
RWR^a^	0.00	10.03	15.19
Molasses	0.55	0.50	0.50
Vitamin-mineral mix	0.53	0.49	0.50
Nutrient content (% of DM)			
Crude protein (CP)	14.62	15.52	15.56
Ether extract (EE)	3.93	3.60	3.22
Ash	9.60	10.10	9.65
Crude fiber (CF)	18.58	24.17	24.39
Nitrogen free extract (NFE)	53.27	46.61	47.17
Neutral detergent fiber (NDF)	49.13	61.67	59.91
Acid detergent fiber (ADF)	34.94	36.03	34.78
Total digestible nutrient (TDN)^b^	66.12	65.40	66.22

^a^Korean rice wine residue; ^b^TDN = 88.936-(0.653*ADF)

Control (Con: 0 % RWR + TMR), Treatment 1 (T1: 10 % RWR + TMR), Treatment 2 (T2: 15 % RWR + TMR)

### Experiment 1: In vitro rumen fermentation

Rumen fluid collected from the rumens of three slaughtered 30-month old Hanwoo steers (700 kg body weight), strained through four layers of surgical gauze, and then pooled in amber bottles with oxygen-free headspace. The bottles were subsequently sealed and immediately transported to the laboratory while maintaining the temperature at 39 °C. Russell and Van Soest [6] buffer solution (pH 7) was mixed with rumen fluid at a 3:1 ratio, after which 100 ml of the mixture was anaerobically dispensed into 160-ml serum bottles under O_2_-free CO_2_. The bottles were then sealed with rubber stoppers and aluminum caps and incubated with shaking (100 rpm) at 39 °C for 0 h, 12 h, 24 h or 48 h. Total gas (TG), pH, NH_3_-N, VFA and other metabolites were analyzed after incubation.

### Experiment 2: Growth performance and blood profiles

A 150-day feeding trial was conducted using 27 Hanwoo steers (6 months old) with body weights of 196 kg ± 8.66 kg. The animals were divided into three treatment groups (nine animals per group) and fed one of the three experimental diets, namely: Control (Con: 0 % RWR + TMR), Treatment 1 (T1: 10 % RWR + TMR), and Treatment 2 (T2: 15 % RWR + TMR). Three cattle were confined in experimental pens (5 m × 10 m) constructed of steel. The cattle house faced south to ensure proper light exposure.

Experimental TMR diets were provided to the animals daily at 2 % of body weight. The total amount of daily feed required was divided into morning (8:00) and afternoon (17:00) feeding. All animals were given free access to fresh drinking water and trace mineral salts (NongHyup, Inc.) throughout the experiment. The experimental cattle were permitted to adapt to the diets for two wk prior to the actual feeding trial. Initial weights of the animals were measured after the acclimatization period, while final body weights were determined and blood samples were collected at the end of the feeding trial.

### Analyses and data collection

Experiment 1- Fermentation parameters were monitored at the end of each incubation time set. pH was measured with a Pinnacle series M530p meter (Schott Instruments, Mainz, Germany) after uncapping each bottle. To measure the total gas, a press and sensor machine was used (Laurel Electronics, Inc., Costa Mesa, CA). Briefly, a needle channel connected to the machine was extended into the sealed fermentation bottle to measure the positive pressure created by the gas build up in the headspace of the bottle at room temperature. A gas flow regulator was subsequently opened, allowing the gas to flow inside a syringe barrel. The plunger was pulled gradually until the pressure reading in the machine display red zero and the volume of gas trapped inside the barrel was recorded as the total gas produced in ml.

Additionally, 1 ml fermenta from each of the serum bottles was immediately centrifuged at 16,609 × *g* for 10 min at 4 °C using a Micro 17TR centrifuge (Hanil Science Industrial Co. Ltd., Korea). The supernatant was kept in 1.5 ml Eppendorf tubes and deep frozen at −80 °C until ammonia-N and VFA analysis. The ammonia-N concentration was measured according to the methods developed by Chaney and Marbach [7] using a Libra S22 spectrophotometer (Biochrom Ltd., CB40FJ, England) at an absorbance of 630 nm. For determination of VFA concentrations, samples contained in Eppendorf tubes were thawed at room temperature, after which they were filtered through 0.2 μm Millipore filters. Standards were made R^2^ at 0.999 prior to sample analysis. Volatile fatty acid concentrations were measured using high performance liquid chromatography (Agilent Technologies 1200 series, Germany) with a UV detector set at 210 nm and 220 nm. MetaCarb 87H (Varian, Germany) column with 0.0085 N H_2_SO_4_ buffer applied at a rate of 0.6 ml/min was used in the determination of fermentation products [8]. The VFA concentration in mM was calculated as parts per million divided by their molecular weight.

Experiment 2- Feed intake and weight gain determination: Voluntary feed intake was calculated as the difference between feed supplied and refusals. Weight gains by the animal (kg) were calculated as the difference between the initial body weights taken before the start of the feeding trial and the final body weights taken at the end of the experiment. Average daily gain (ADG) was calculated as weight gained in kilograms divided by the experimental period (150 d). Feed efficiency (FE) was calculated as the ratio of weight gain to the amount of feed consumed. At the end of the experiment period, five ml blood samples were collected from the jugular vein of the animals into sterilized vacuum tubes (Green Cross MS, Korea) containing K3-EDTA. The tubes were gently inverted several times, kept in an ice box, and then centrifuged for 15 min at 890 × g at 4 °C, after which they were stored for eight h in a refrigerator at 4 °C prior to separation and analysis of the serum. The plasma was also transferred to a storage tube and labeled with the date and animal identification, then analyzed fresh or stored at −20 °C until analysis. Total alcohol, protein, albumin, creatinine, blood urea nitrogen and glucose concentrations were analyzed using an automatic blood analyzer (Express Plus, Ciba-Corning, CA, USA). Serum glutamic oxaloacetic transaminase (SGOT), serum glutamic pyruvic transaminase (SGPT), total cholesterol, high density lipoprotein (HDL), low density lipoprotein (LDL) and triglyceride concentrations in the blood samples were analyzed by Green Cross Corp., Gyeonggido, Korea.

### Statistical analysis

Data were analyzed by analysis of variance (ANOVA) using the general linear model (GLM) for a randomized complete block design. All treatments were conducted in triplicates. Duncan’s Multiple Range Test and Orthogonal Polynomial Contrast were used to identify differences between and among treatments and control. A *P <* 0.05 was considered to indicate statistical significance. All analyses were carried out using Statistical Analysis Systems (SAS) version 9.1 [9].

## Results

### Rumen fermentation characteristics

Inclusion of RWR resulted in non-significant total gas production between control and treatment groups, with 120.67 ml to 122.00 ml and 150.33 ml to 166.33 ml of gas being produced at 24 h and 48 h, respectively. Similarly, pH was higher (*P <* 0.05) in control than the treatment groups after 12 h of incubation, but non-significant values ranging from 6.23 to 6.30 at 24 h, and 6.16 to 6.18 at 48 h were observed. Ammonia nitrogen (NH_3_-N) concentrations were significantly higher in control and T1 than T2 after 12 h of incubation and the concentrations became comparable after 24 h and 48 h of incubation (Table 2).

**Table 2:** In vitro rumen fermentation parameters as affected by the addition of Korean rice wine residue (RWR)

Parameters	Incubation	Con	T1	T2	SEM	*P* value	
	time (h)					All	C vs T
Total gas (ml)	0	62.67	65.00	64.33	1.181	0.460	0.248
	12	104.00	100.67	101.33	1.264	0.312	0.149
	24	122.00	120.67	121.00	1.953	0.884	0.643
	48	150.33	155.00	166.33	10.630	0.608	0.479
pH	0	6.60	6.62	6.64	0.014	0.163	0.101
	12	6.43^a^	6.38^ab^	6.28^c^	0.033	0.046	0.049
	24	6.23	6.27	6.30	0.050	0.655	0.411
	48	6.18	6.18	6.16	0.044	0.949	0.876
Ammonia Nitrogen (mM)	0	3.39	3.41	3.44	0.490	0.998	0.956
	12	5.77^a^	5.26^a^	3.65^b^	0.281	0.029	0.031
	24	7.65	6.09	6.19	0.887	0.598	0.340
	48	13.54	11.25	12.56	1.362	0.586	0.408

Values are shown as mean. Means for the same period (0 h, 12 h, 24 h, or 48 h) marked with the same letters (a > b > c) did not differ significantly within treatments (*P* < 0.05), as determined by Duncan's multiple range test. SEM is the standard error mean. C vs T is the comparison between control and treatments. Control (Con: 0 % RWR + TMR), Treatment 1 (T1: 10 % RWR + TMR), Treatment 2 (T2: 15 % RWR + TMR)

At 12 h and 24 h, the acetate concentrations were higher (*P <* 0.05) in the control (31.74 mM and 38.05 mM) and T1 (28.52 mM and 36.04 mM) than T2 (26.64 mM and 32.54 mM). Additionally, propionate concentration was higher (*P <* 0.05) in the control (18.44 mM) and T1 (16.49 mM) than T2 (12.60 mM) while butyrate concentration was highest (*P <* 0.05) in T2 by 6.81 mM and 4.82 mM relative to the control and T1, respectively after 24 h of incubation (Table 3). Total VFA concentration was highest (*P <* 0.05) in the control, followed by T1 and T2 with concentrations of 69.95 mM, 63.35 mM, and 60.78 mM, respectively at 24 h. Except for the 24 h incubation time, the VFA concentration did not differ significantly among groups.

**Table 3:** In vitro volatile fatty acid concentration as affected by the addition of Korean rice wine residue (RWR)

Parameters	Incubation time (h)	Con	T1	T2	SEM	*P* value	*P* value
						All	C vs T
Acetate	0	23.88	23.56	23.57	0.163	0.075	0.081
(mM)	12	31.74^a^	28.52^ab^	26.64^b^	1.348	0.101	0.050
	24	38.05^a^	36.04^ab^	32.54^b^	1.310	0.075	0.067
	48	41.81	41.21	40.77	0.361	0.387	0.224
Propionate	0	5.09	5.10	5.24	0.059	0.238	0.199
(mM)	12	13.87	11.06	9.79	1.238	0.169	0.080
	24	18.44^a^	16.49^a^	12.60^b^	0.558	0.017	0.016
	48	20.64	19.14	19.92	0.574	0.297	0.191
Butyrate	0	ND	ND	ND	ND	ND	ND
(mM)	12	8.51^a^	7.38^ab^	5.71^b^	0.568	0.062	0.051
	24	8.83^b^	10.82^b^	15.64^a^	1.060	0.015	0.021
	48	11.74	12.12	15.28	1.735	0.404	0.430
Total VFA	0	28.97	28.67	28.81	0.087	0.269	0.236
(mM)	12	54.13	46.96	42.15	2.699	0.068	0.017
	24	69.95^a^	63.35^b^	60.78^b^	1.569	0.014	0.030
	48	74.19	72.47	75.98	2.379	0.578	0.761
A:P	0	4.69^a^	4.62^ab^	4.50^b^	0.025	0.001	0.002
	12	2.33	2.64	2.73	0.155	0.243	0.302
	24	2.32	2.21	2.59	0.105	0.126	0.057
	48	2.03	2.15	2.05	0.052	0.409	0.330

Values are shown as mean. Means for the same period (0 h, 12 h, 24 h, or 48 h) marked with the same letters (a > b > c) did not differ significantly within treatments (*P* < 0.05), as determined by Duncan's multiple range test. SEM is the standard error mean. C vs T is the comparison between control and treatments. Control (Con: 0 % RWR + TMR), Treatment 1 (T1: 10 % RWR + TMR), Treatment 2 (T2: 15 % RWR + TMR)

### Growth performance and blood profiles

The feed cost was 8 % and 15 % lower in T1 and T2 compared to control. Table 4 shows the growth performance and blood profiles of Hanwoo steers fed with TMR mixed with or without RWR. T1 had the highest total weight gain (*P* = 0.013) and ADG (*P* = 0.012), with values significantly higher (*P <* 0.05) than the control by 14.67 kg and 0.10 kg/d, respectively. However, differences in these growth performance parameters between T1 and T2 and between T2 and the control were found to be comparable. Moreover, the blood profile of animals after the feeding trial revealed that no ethanol was present in the blood of any animals used in the experiment, while values for albumin, SGPT, SGOT, glucose, total cholesterol, triglycerides and LDL were comparable between all groups. Highest HDL and total blood protein concentration were detected in T2 (108.44 g/dl and 6.61 g/dl) followed by the control (99.33 g/dl and 6.39 g/dl) and T1 (96.56 g/dl and 6.19 g/dl). Relative to the control, BUN levels were significantly higher in T1 (by 2.32 mg/dl) and significantly lower in T2 (by 3.10 mg/dl). Furthermore, the creatinine value for T2 was 0.17 mg/dl lower than the comparable values for the control and T1.

**Table 4:** Growth performance and blood profiles of Hanwoo steers fed with or without Korean rice wine residue

Parameters	Con	T1	T2	SEM	*P* value	
					All	C vs T
Growth performance						
Total gain (kg)	153.33^b^	168.00^a^	160.83^ab^	2.816	0.013	0.009
ADG (kg/d)	1.00^b^	1.09^a^	1.04^ab^	0.018	0.012	0.008
Feed intake	9.81	9.70	9.90	0.105	0.04	0.845
Feed efficiency	0.102	0.112	0.105	0.002	0.053	0.082
Blood profiles						
Ethanol (%)	0	0	0	0	-	
Albumin (g/dL)	3.68	3.66	3.87	0.088	0.202	0.45
AST/SGOT (U/L)	66.78	59	59.89	3.406	0.246	0.099
ALT/SGPT (U/L)	26.89	23.89	24.00	1.673	0.374	0.165
Glucose (mg/dL)	63.00	60.56	69.56	3.044	0.124	0.591
T. Chol (mg/dL)	114.22	110	123.33	6.248	0.358	0.764
Triglyceride (mg/dL)	26.11	25.44	30.44	2.571	0.356	0.572
LDL (mg/dL)	17.33	14.89	18.44	1.202	0.124	0.655
HDL (mg/dL)	99.33^b^	96.56^b^	108.44^a^	5.804	0.369	0.675
Creatinine (mg/dL)	0.80^b^	0.80^b^	0.97^a^	0.047	0.028	0.178
BUN (mg/dL)	14.14^b^	16.47^a^	11.04^c^	0.755	0	0.679
Total protein (g/dL)	6.39^ab^	6.19^b^	6.61^a^	0.107	0.04	0.935

Values are shown as mean. Means for the same period (0 h, 12 h, 24 h, or 48 h) marked with the same letters (a > b > c) did not differ significantly within treatments (*P* < 0.05), as determined by Duncan's multiple range test. SEM is the standard error mean. C vs T is the comparison between control and treatments. Control (Con: 0 % RWR + TMR), Treatment 1 (T1: 10 % RWR + TMR), Treatment 2 (T2: 15 % RWR + TMR)

## Discussion

### In vitro rumen fermentation characteristics

The results of in vitro ruminal fermentation revealed that the replacement of RWR to TMR did not cause a significant change in total gas production as well as pH level. However, total gas production increased while pH level decreased with advancing rumen fermentation period of the substrate. Higher gas production could be due to high contents of easily fermentable starches, sugars or hemicelluloses as substrate to rumen microbes [10]. Mamuad et al. [11] added that NFE, total digestible nutrient (TDN), and ruminal fermentation have a directly proportional relationship. High NFE contents of RWR (Table 1) replaced in TMR corroborate high ruminal fermentation which increases total gas production as incubation time became longer and resulted in reduced pH.

High NH_3_-N levels indicate that the soluble fraction of protein is also high, indicating greater catabolism of protein and non-protein nitrogen (NPN) [12]. The ideal concentration of rumen NH_3_-N for an efficient digestion has been estimated to be 3.56 mM to 4.99 mM [13]. Perdock [14] reported that the optimal rumen NH_3_-N concentrations was about 14.27 mM. The values obtained for the RWR treatments were higher than those reported by Satter and Slyter [13], but lower than those reported by Perdock [14].

Volatile fatty acid, which is the first source of energy for ruminant animals, is influenced by the feed quality, quantity, and allowance method. McDonald et al. [15] reported that the total concentration of VFAs varies widely according to diet and time elapsed since the previous meal, although it is normally in the range of 70 mM to 150 mM. In this study, VFA increased gradually with increased incubation periods, and the total concentrations of VFAs were comparable among control and treatment groups after 48 h of incubation. Feeds high in rapidly fermentable carbohydrates lead to populations of bacteria which produce relatively more propionate and butyrate than acetate [16], that indicate significantly higher butyrate concentration in T2. This indicates that replacement of RWR increased the butyrate concentration, which possessed important function in the intestinal epithelium. Van Nevel and Demeyer [17] stated that when soluble carbohydrates and starch-rich diets are fed to ruminants, the production of propionate increases. However, lower NFE in treatment groups were observed and hence, explained the lower propionate concentration after 24 h of incubation. Nevertheless, comparable propionate concentrations were observed among control and treatment groups were observed after 48 h of incubation.

Upon evaluation of the VFA concentrations, the ratio of acetic:propionic acid (A:P ratio) reflects rumen fermentation. When rumen fermentation conditions are optimal, the A:P ratio should be greater than 2.2 [15], which corroborates this study. Lower propionate and acetate concentrations in T2 after 24 h of incubation can be associated with the diet given. A higher level of fermentable carbohydrate in the diet could lead to a higher level of propionic acid, and thus reduced fiber digestion and possibly acidosis. High levels of acetic acid can indicate a high fiber, low fermentable carbohydrate diet [15]. In the present study, the A:P ratio ranged from 2.03 to 2.15, but was not significantly different after 48 h of incubation.

### Growth performance and blood profile characteristics

#### Growth performance

Hanwoo steers fed with 10 % RWR had higher total weight gain, ADG and FE, which means replacement of RWR increased its growth performance. This result was in concordance with Lin et al. [18] data observed when they fed the steers with alcohol-fermented feed diet at growing stage. The steers grew significantly (*P <* 0.05) faster than those fed the control diet. The significant increase (*P <* 0.01) in daily gain in the treatment groups was due to the presence of wet distillers grains (WDG) in the diet, which was comparably with the results reported by Firkins et al. [19]. In addition, other by product used as feed alternative such as brewer’s grains has also been reported to produce favorable performance in dairy cows [20] growing calves [21]. National Research Council [22] stated that the CP content of RWR was very similar to that of brewer’s dried grains with 28.67 % vs 29.10 %. Therefore, improved performances in Hanwoo steers were observed when RWR was used as feed alternative.

#### Blood metabolites

As mentioned above, RWR is a by-product of Korean rice wine, and the lack of ethanol in the blood profiles is a good indication that the alcohol content from the residue did not affect the growing Hanwoo steers. Blood albumin concentration was previously reported to decrease with increasing synthesis of muscle proteins [23]; however, no such correlation was observed in the present study. Korean rice wine residue treatment groups achieved a better performance than the control group, consistent with the above statements. A lower SGOT and SGPT were obtained in RWR supplemented growing cattle, and Kim et al. [24] found that the low levels of SGOT and SGPT were favorable for healthy cattle. Serum proteins are believed to be indicative of the nutritional status of the animal, and constitute a portion of the amino acid pool of the body [25]. Biochemically, the higher blood total protein and albumin concentration in both treatments were in the normal range [26]. BUN in cattle was affected by dietary levels of protein and energy [27], hence lower BUN was observed in T2. Also, the lower correlation between serum BUN and protein in steers fed fermented feed could result from the increase in BUN levels and the reduction in protein synthesis, and vice versa [28]. The rumen microflora metabolizes a larger percentage of urea than the enteric flora in monogastric animals, often preventing BUN concentrations being increasing proportionately with creatinine levels in cattle [29].

In the present study, the creatinine concentration in calves fed the basal diet alone was below the normal range. Lin et al. [18] reported that providing alcohol-fermented feed to ruminants increased the propionate, triglyceride, cholesterol and glucose concentrations in the blood with increasing weight gain. The findings of this experiment did not differ with regard to LDL concentrations among the control and treatment groups. However, the HDL concentrations of T2 differed significantly among the control and treatment groups. Body fat is correlated with HDL and LDL concentrations, with increases in body weight or fat resulting in increased HDL and LDL levels [24]. The results of the present study revealed similar LDL levels in the control and treatment, which were favorable for animals and similar to the views described above.

## Conclusion

These results suggest that TMR amended with 15 % RWR can be used as an alternative feed resource for ruminants to reduce feed cost while increasing ADG and total gain.
